# Development of a gastroschisis core outcome set

**DOI:** 10.1136/archdischild-2017-314560

**Published:** 2018-03-14

**Authors:** Benjamin Saul Raywood Allin, Nigel J Hall, Andrew R Ross, Sean S Marven, Jennifer J Kurinczuk, Marian Knight

**Affiliations:** 1 National Perinatal Epidemiology Unit, University of Oxford, Oxford, UK; 2 Southampton Children’s Hospital, Southampton, UK; 3 Oxford Children’s Hospital, Oxford, UK; 4 Sheffield Children’s Hospital, Oxford, UK

**Keywords:** gastroschisis, paediatric surgery, epidemiology, core outcome sets

## Abstract

**Objective:**

Outcome reporting heterogeneity impedes identification of gold standard treatments for children born with gastroschisis. Use of core outcome sets (COSs) in research reduces outcome reporting heterogeneity and ensures that studies are relevant to patients. The aim of this study was to develop a gastroschisis COS.

**Design and setting:**

Systematic reviews and stakeholder nomination were used to identify candidate outcomes that were subsequently prioritised by key stakeholders in a three-phase online Delphi process and face-to-face consensus meeting using a 9-point Likert scale. In phases two and three of the Delphi process, participants were shown graphical and numerical representations of their own, and all panels scores for each outcome respectively and asked to review their previous score in light of this information. Outcomes were carried forward to the consensus meeting if prioritised by two or three stakeholder panels in the third phase of the Delphi process. The COS was formed from outcomes where ≥70% of consensus meeting participants scored the outcome 7–9 and <15% of participants scored it 1–3.

**Results:**

71 participants (84%) completed all phases of the Delphi process, during which 87 outcomes were assessed. Eight outcomes, mortality, sepsis, growth, number of operations, severe gastrointestinal complication, time on parenteral nutrition, liver disease and quality of life for the child, met criteria for inclusion in the COS.

**Conclusions:**

Eight outcomes have been included in the gastroschisis COS as a result of their importance to key stakeholders. Implementing use of the COS will increase the potential for identification of gold standard treatments for the management of children born with gastroschisis.

**What is already known on this topic?**

- It is not currently possible to identify gold standard treatments for children with gastroschisis, partly because of outcome reporting heterogeneity.
- Many gastroschisis studies investigate outcomes that are not relevant to patients or clinical practice.
- The use of core outcome sets in research reduces outcome reporting heterogeneity and helps improve the clinical relevance of research.

**What this study adds?**

- This study has developed a gastroschisis core outcome set consisting of eight outcomes that are important to parents, people born with gastroschisis and clinicians.
- The eight outcomes are death, sepsis, growth, number of operations, time on parenteral nutrition, liver disease, number of severe gastrointestinal complications and quality of life.
- The core outcome set can be used in future observational and interventional studies and will reduce outcome reporting heterogeneity and increase clinical relevance of studies.

## Introduction

Gastroschisis is increasing in incidence and is estimated to affect between 3.6 and 4.4 per 10 000 live births in the UK.^1 2^ As with many neonatal surgical conditions, there are a number of treatment options in everyday use, and for gastroschisis, the two most common are operative primary fascial closure and silo placement, followed by staged reduction and delayed closure. Strategies for immediate postoperative management, introduction of enteral feeding and parenteral nutritional support also vary widely. There is therefore robust debate among the paediatric surgical and neonatal communities as to which intervention or combination of interventions produces the best outcomes, and due to limitations with the primary evidence base, it is not currently possible for systematic reviews to reliably inform this debate. Limitations of the primary evidence base include the small sample size and retrospective nature of many of the studies and the existence of significant outcome reporting heterogeneity.^3 4^ Outcome reporting heterogeneity suggests there is a lack of consensus among researchers as to which outcomes should be used to define success of treatment in a particular condition and indicates that studies are at risk of lacking relevance to patients, of being affected by reporting bias and being difficult to meta-analyse.

A core outcome set (COS) is a group of outcomes that have been identified by key stakeholders as being the most important in determining success of treatment of a particular condition. Once a COS has been developed for a particular condition, all future studies conducted within the scope of the COS should investigate and report as a minimum all outcomes included within the COS.^5^ Additional outcomes can also be investigated and reported if appropriate for the study, but the reporting at a minimum of all core outcomes ensures that a study will be relevant to patients and clinical practice, at a low risk of reporting bias and meta-analysable with other studies investigating the same clinical question.^5^ The aim of this study was therefore to develop a COS that could be used in studies comparing the overall success of postnatal treatments for children born with gastroschisis.

## Methods

### Protocol registration

The protocol was prospectively registered in October 2014 on the Core Outcome Measures in Effectiveness Trials (COMET) initiative website (http://www.comet-initiative.org/studies/searchresults?guid=d1e190c8-a2eb-4d49-a341-7d3ec79be12c) and published in a peer-reviewed journal.^6^

### Scope

The COS is intended for use in studies comparing postnatal interventions for the treatment of children born with gastroschisis in high-income countries. It is likely that outcomes of importance in low-income and middle-income countries will be different to those that are important in high-income settings, and therefore, the relevance of the COS to studies conducted in these settings should be considered prior to it being used. The COS is also not intended to be applicable to studies investigating antenatal interventions or factors related to the mode or timing of delivery of babies with a prenatal diagnosis of gastroschisis.

### Process

**Figure 1:**
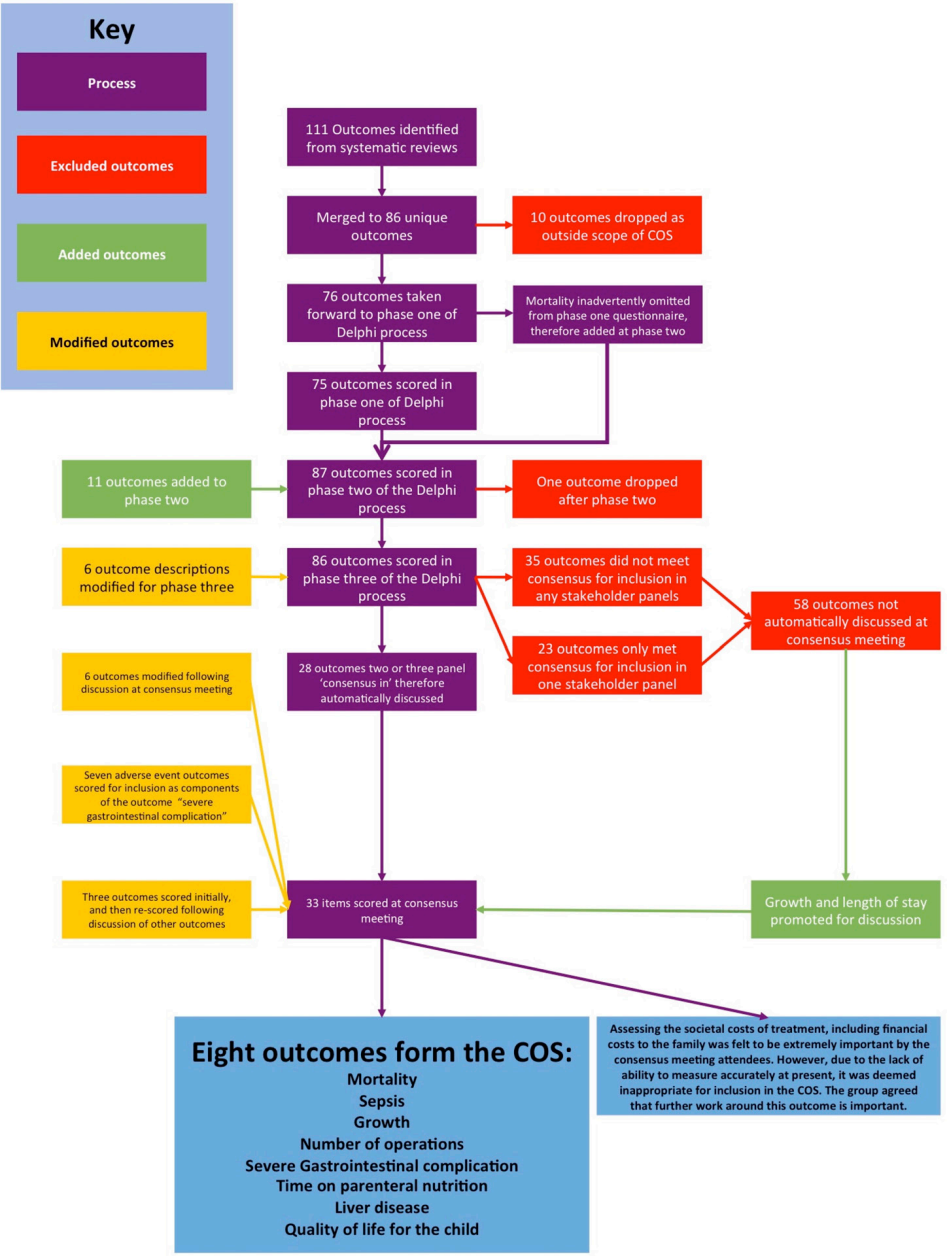
Summary of the gastroschisis core outcome set (COS) development process.

Three panels of stakeholders completed a three-phase online Delphi process in order to prioritise outcomes identified from a systematic review and stakeholder nomination. Prioritised outcomes were discussed and rescored at a face-to-face consensus meeting, and those that met a prespecified threshold were included in the final COS. A separate face-to-face meeting was held to identify measurement definitions for each outcome included in the COS (figure 1).

### Participants

So as to represent the full spectrum of clinical and personal experience of gastroschisis, participants were recruited across a range of clinical specialties involved in the treatment of children born with gastroschisis and also from families where one or more people had been born with gastroschisis. Experts were recruited according to the strategies described in table 1 with those selected to participate asked to nominate additional potentially eligible stakeholders. Clinical stakeholders were recruited only from the UK in order to ensure funding could be used to maximise the number of participants attending the consensus meeting while also maintaining the meeting attendee’s representativeness of the wider study participants. Prior to inclusion in the study, experts were asked to register their interest via a customised website, where details were collected documenting their experience of gastroschisis. Registrations were reviewed by the Study Management Group (SMG) to ensure that participants had sufficient expertise in gastroschisis management or lived experience of gastroschisis to participate in the study.

**Table 1:** Stakeholder recruitment strategies

Stakeholder group	Panel	Recruitment methods
People born with gastroschisis	Personal experience panel	Mailing lists, websites and Facebook groups of UK and international gastroschisis support groups.
Parents of children born with gastroschisis		Mailing lists and meetings for a Parental Advisory Group established by the National Perinatal Epidemiology Unit.
Paediatric surgeons	Neonatal panel	Direct approaches to experts known to members of the SMG and those identified on a search of the British Association of Paediatric Surgeons (BAPS) register as having a special interest in management of children with gastroschisis. BAPS mailing lists, newsletters and website. Direct approach to clinical leads at each paediatric surgical centre in the UK for the BAPS-CASS gastroschisis study.
Neonatologists	Neonatal panel	Direct approach to experts known to members of the SMG. Mailing lists, bulletin and website of the British Association of Perinatal Medicine.
Fetal medicine specialists	Neonatal panel	Direct approach to experts known to members of the SMG. Mailing list of the fetal medicine clinical study group of the British Maternal and Fetal Medicine Society.
Specialist nurses	Neonatal panel	Direct approaches to experts known to members of the SMG. Mailing list of the Neonatal Nurses Association and the National Neonatal Surgical Benchmarking Group.
Paediatricians	Non-neonatal panel	Direct approach to experts known to members of the SMG. Mailing list of the British Society of Paediatric Gastroenterology Hepatology and Nutrition.
Researchers	Non-neonatal panel	Direct approaches to experts known to members of the SMG and prominent gastroschisis researchers identified through searches of the literature.
Specialist paediatric surgical nurses	Neonatal panel	Direct approach to experts known to members of the SMG.
Dietitians	Non-neonatal panel	Direct approach to experts known to members of the SMG. Mailing list of the British Society of Paediatric Gastroenterology Hepatology and Nutrition.

BAPS-CASS, British Association of Paediatric Surgeons Congenital Anomalies Surveillance System; SMG, Study Management Group.

Data presentation throughout the Delphi process was simplified by combining stakeholder groups into a neonatal panel, a non-neonatal panel and a personal experience panel as described in table 1, within which, opinions were anticipated to be broadly similar.

### Information sources

Two systematic reviews, each with a prospectively registered protocol, were conducted by separate groups who had each set out to develop a COS for use in determining the overall success of treatment for a child born with gastroschisis. The first of these reviews was a broad, scoping review, including all comparative study designs,^7^ while the second focused solely on randomised controlled trials and systematic reviews.^8^ Outcomes identified from the systematic reviews were assessed by the SMG and mapped to unique terms. Outcomes assessing the success of antenatal interventions were dropped as they were outside of the scope of the COS.

In phase one of the Delphi process, stakeholders were asked to propose additional outcomes that they felt were important but had not been identified by the systematic reviews. These outcomes were reviewed by the SMG, and if within the scope of the COS, were taken forward for assessment in phase two of the Delphi process. See online supplementary material 1 for list of all outcomes considered at any stage.

10.1136/archdischild-2017-314560.supp1 Supplementary file 1

### Dropping and modification of outcomes

No outcomes were dropped between phase one and phase two of the Delphi process. Between phase two and phase three, outcomes were dropped if ≥50% of participants in all panels scored them 1–3 and <50% of participants in any panel scored them 7–9. Outcomes were automatically discussed at the consensus meeting if in phase three of the Delphi process two or more panels deemed them to meet the threshold for inclusion in the COS. As per guidance from the COMET initiative, the threshold for inclusion in the COS was defined as ≥70% of participants scoring an outcome of 7–9, and <15% scoring 1–3.^9^ Other outcomes were only discussed and rescored at the consensus meeting if there was unanimous agreement among the meeting attendees that they warranted further discussion.

Comments were sought from participants in relation to clarity of outcome descriptions throughout each phase of the Delphi process. All comments were reviewed by the SMG, and if necessary, outcome descriptions/terminology were modified to improve their clarity and understanding.

### Consensus definition

Outcomes were deemed to have met consensus for inclusion in the COS if ≥70% of participants at the consensus meeting scored them 7–9 and <15% scored them 1–3.

### Assessment of attrition bias

Median phase one scores for the outcomes included in the COS (or their nearest approximation where the outcome was added after phase one) were compared between participants within each panel who completed all three phases of the Delphi process and those who only completed phase one. Wilcoxon rank-sum test was used to compare scores, and in order to reduce the risk of a type I error, the Bonferroni correction was used to set the level of significance at a P value of <0.002.

## Results

### Protocol modifications

It was initially proposed that people born with gastroschisis and parents of children born with gastroschisis should only be recruited from the UK. However, despite extensive work with gastroschisis charities, it proved difficult to recruit to these stakeholder groups when participation was limited to the UK. It was therefore decided by the SMG that it was more important to ensure a strong voice of personal experience throughout the study than it was to ensure that it was feasible for all participants to have the opportunity to attend the consensus meeting, and recruitment was therefore expanded to include people with personal experience of gastroschisis who were treated in other high-income countries. Participants in other stakeholder groups were still restricted to those based in the UK.

It was initially proposed that no outcomes would be dropped between phases of the Delphi process. However, after publication of the protocol, but prior to phase one of the Delphi process, it was decided by the SMG that in order to allow participants to focus on outcomes likely to be of greater importance, they should instead be retained as described in the *dropping and modification of outcomes section*.

### Participants

One hundred and sixty-four people registered to participate in the Delphi process, 102 (62%) of whom completed phase one. Eighty-five (83%) of 102 eligible participants then completed phase two, and 71 (84%) of 85 eligible participants completed phase three (table 2).

**Table 2:** Study participants

Number of participants						
	Registering for round one	Completing round one (% of those eligible)	Completing round two (% of those eligible)	Completing round three (% of those eligible)	Consensus meeting	Measurement meeting
Neonatal panel	58	52 (90)	47 (90)	43 (91)	15	10
Non-neonatal panel	8	8 (100)	7 (88)	6 (86)	4	3
Personal experience panel	98	42 (43)	31 (74)	22 (71)	5	1
Total	164	102 (62)	85 (83)	71 (84)	24	14

Of the 71 participants that completed all three phases of the Delphi process, 19 (27%) were paediatric surgeons, 13 (18%) were neonatologists, 11 (16%) were specialist nurses, 2 (3%) were paediatric gastroenterologists, 2 (3%) were paediatric dieticians and 22 (31%) were parents of children born with gastroschisis. Fourteen (64%) of the parents who completed all three phases of the Delphi process had children born with gastroschisis who were less than 5 years of age at the time of the study, four (18%) had children between 5 and 10 years of age, two (1%) had children over 10 years of age and two (1%) preferred not to say how old their child was.

### Outcomes

Following review by the SMG and removal of outcomes outside the scope of the COS, 75 outcomes were carried forward from the systematic reviews to phase one of the Delphi process. Twelve additional outcomes were proposed during phase one, leading to assessment of 87 outcomes in phase two, 86 (99%) of which were carried forward to phase three (online supplementary material 1). Following scoring in phase three, 28 outcomes (33%) met the criteria for automatic discussion at the consensus meeting, with two additional outcomes discussed following unanimous agreement by the meeting attendees that they warranted further review (table 3). Eight outcomes (box 1 and table 4) met the criteria for inclusion in the COS, with the additional outcome *societal cost (including financial cost to the family)* noted as important by the meeting attendees but not included within the COS due to the lack of ability to accurately measure such an outcome at present.

**Table 3:** Outcomes discussed at the consensus meeting, categorised into OMERACT filter 2.0 core areas

Mortality outcomes	Life impact outcomes	Pathophysiological manifestation outcomes	Resource utilisation outcomes	Adverse event outcomes
Mortality	Home parenteral nutrition	Quality of life for the child	Short bowel syndrome	Abdominal compartment syndrome†
	Need for total parenteral nutrition postdischarge	Bowel lengthening procedure required	Cholestasis	Bowel ischaemia
	Reoperation	Time on total parenteral nutrition	Unspecified measures of growth*	Bowel obstruction†
	Societal costs, including financial costs for the family	Time on parenteral nutrition		Bowel resection†
	Rehospitalisation	Liver transplant		Intestinal perforation†
	Length of stay*	Small bowel transplantation		Necrotising enterocolitis†
		Need for a permanent stoma		Infection with systemic sequelae
		Chronic GI symptoms		Anastomotic stricture
		Gastrointestinal dysfunction		Gastrointestinal complication†
		Neurodevelopmental outcomes		Intestinal failure associated liver disease

*Outcomes not meeting criteria for automatic discussion at the consensus meeting and only promoted after unanimous agreement by the meeting attendees.

†Outcomes combined to the composite outcome severe gastrointestinal complication.

GI, gastrointestinal.

**Table 4:** Outcomes meeting consensus for inclusion in the NETS^1G^ core outcome set

Core outcome	Score 7–9 (%)	Reporting time-points
Death	100	Cohort or intervention study time-points
Sepsis	100	Cohort or intervention study time-points
Growth	100	Cohort or intervention study time-points
Number of operations	100	Cohort or intervention study time-points
Severe gastrointestinal complication	96	Cohort or intervention study time-points
Time on parenteral nutrition	87	Cohort study time-points only
Liver disease	74	Cohort or intervention study time-points
Quality of life for the child	73	Cohort or intervention study time-points

NETS, Next Stage in Evidence-based paediatric surgical Treatment Strategies 1 - Gastroschisis.

### Definition and measurement of outcomes

A literature review informed by a previously published systematic review^7^ identified existing definitions, measurement tools and common measurement time-points for outcomes included in the COS. The 14 attendees at the measurement meeting were asked to review summaries of this literature in advance of the meeting in order to guide discussion among the group. Following discussion, unanimous agreement was reached on definitions and methods of measurement for each core outcome (box 1). Appropriate time-points for reporting these core outcomes were also discussed, and it was unanimously agreed that these should be kept as close as possible to standard time-points for reporting surgical and paediatric outcomes (table 5). In order to make future meta-analysis more meaningful, studies using the developed COS should report outcomes at at least one of these time points. Further rationale for selection of these time-points, and for using different time-points for intervention and cohort studies is described in detail in a previously published Hirschsprung’s disease COS development study.^10^

**Table 5:** Proposed time-points for measurement of core outcomes

Cohort studies	Intervention studies
28 days of age	30-day postintervention
	90-day postintervention
One year of age	One-year postintervention
Five years of age	Five-year postintervention
Ten years of age	Ten-year postintervention
Every subsequent 10 years	Every subsequent 10 years

### Attrition bias

Median phase one scores for the eight outcomes included in the COS, or their nearest approximations, were compared between participants who completed all three phases of the Delphi process and those who completed phase one only. No statistically significant differences were seen between any of these groups (table 6).

Box 1 **The gastroschisis core outcome set**

**Death**

- Number (%) of infants who have died.

**Sepsis**

- Median (IQR and range) number of times treatment was given for proven or presumed sepsis.
- Number of episodes where sepsis was proven by a positive blood or CSF culture should be reported separately from the number of episodes where treatment was given for sepsis, but the blood or CSF culture was negative.

**Growth**

- Median (IQR and range) z-score for weight, length and head circumference in studies reporting outcomes at or prior to 1 year of age.
- Median (IQR and range) z-score for weight and height in studies reporting outcomes after 1 year of age.

**Number of operations**

- Median (IQR and range) number of operations per infant.
- The type of operations undertaken should be categorised according to whether they were performed under general or local anaesthetic and whether they were an abdominal operation, central venous catheter insertion or ‘other’ operation. ‘Other’ operations should only be reported when performed under general, not local, anaesthetic.
- Each episode of silo placement, replacement and reapplication should be reported as a separate abdominal operation, with abdominal closure reported separately to silo placement.

**Severe gastrointestinal complication**

- Median (IQR and range) number of severe gastrointestinal complications per infant.
- Severe gastrointestinal complication *only includes*:
  - intestinal perforation
  - any intestinal resection, regardless of amount of bowel removed or the indication for the resection
  - mechanical intestinal obstruction resulting in a repeat laparotomy
  - abdominal compartment syndrome*
  - enterocolitis.†

**Time on parenteral nutrition**

- Median (IQR and range) number of days *any* parenteral nutrition was received per infant in studies reporting outcomes at or prior to 1 year of age.
- Number (%) of infants receiving *any* parenteral nutrition in studies reporting outcomes after 1 year of age.

**Liver disease**

- Number (%) of infants with persistent conjugated hyperbilirubinaemia (>50 μmol/L) for ≥2 weeks with no known other underlying liver disease.

**Quality of life for the child**

- Median (IQR and range) PedsQL score in each study group.
- If appropriate, the median (IQR and range) score from the PedsQL gastrointestinal symptoms and family impact modules in each study group should also be reported.

*Defined as ‘suspected raised intra-abdominal pressure with at least two of oliguria or anuria, respiratory de-compensation, hypotension/shock, or metabolic acidosis, that leads to intervention’.†Defined as ‘suspected enterocolitis with at least one of bilious aspirates or emesis, abdominal distension or occult or gross blood in stool (no fissure), and at least one of pneumatosis intestinalis, hepatobiliary gas, pneumoperitoneum’.CSF, cerebrospinal fluid; PedsQL, pediatric quality of life inventory.

## Discussion

Using robust consensus methodology, we have enabled key stakeholders to identify eight outcomes as being the most important in determining the overall success of treatment of a child born with gastroschisis. These are: death, sepsis, growth, number of operations, severe gastrointestinal complication, time on parenteral nutrition, liver disease and quality of life for the child. By developing the COS using Delphi methodology combined with detailed discussion of outcomes at a consensus meeting, we anticipate that the included outcomes are relevant to clinical practice, parents and patients and appropriate for differentiating the relative merits of gastroschisis treatments. Furthermore, the COS has been designed to be practical to use in multiple study designs. The number of outcomes is relatively small and each has been robustly defined, with an appropriate measure and time-point for reporting identified. This level of detail should enable the immediate practical implementation of the COS.

A particular strength of this process has been the involvement of a wide range of stakeholder groups including parents of children born with gastroschisis. Unfortunately, however, there were no individuals born with gastroschisis who completed all three phases of the study. The difficulty engaging adults treated for gastroschisis as a child may be due to the fact that many have no ongoing active involvement with medical services or charities. It is difficult to know if this population of adults do not have regular contact with medical services because they are symptom free or because they have become detached from these services. If the former is true, the COS will likely remain fully representative of the outcomes that are important in determining treatment success, as the majority of ‘experience’ of gastroschisis will be from the parental point of view. However, if it is the latter, and there are differences in opinion of which outcomes are important between parents, and adults who were treated for gastroschisis as a child, then the COS may under-represent the treated adult’s opinion.

In addition to the difficulties that were experienced recruiting people born with gastroschisis to the personal experience panel, the attrition rate in this panel was also higher than in the two other panels. However, this was still in line with other published studies.^11^ It is unclear why the attrition rate was higher, but we would speculate that the demographics of those who are likely to be members of the personal experience panel might have influenced their ability to find time to complete all three phases of the study. There were, however, no differences identified in scoring patterns between those participants who completed all three phases of the Delphi process and those who only completed phase one, and we therefore do not believe that the identified attrition will have affected the results of the process.

Currently, many COS development processes conduct interviews with non-medical participants prior to starting the Delphi process. These interviews are used to identify outcomes that are important to patients but not reported by the existing literature. In this COS development process, we opted not to conduct interviews and instead gave participants the opportunity to propose new outcomes in phase one of the Delphi process. This decision was based on our experience of developing a Hirschsprung’s disease COS,^10^ where analysis of the additional outcomes proposed by participants, and comments left during the Delphi process reassured us that the likelihood of missing important outcomes by not conducting qualitative interviews prior to starting the Delphi process was low. In addition, we believed there to be significant benefit to not conducting qualitative interviews in that it reduced the cost and time necessary to develop the COS and therefore increased the efficiency with which its use could be implemented in future research. Interestingly, despite some methodological differences, there is overlap between the outcomes included in this COS, outcomes included in the recently developed Hirschsprung’s disease COS^10^ and a paediatric asthma COS.^12^ All three have included death and quality of life, as well as a measure of the need for repeated medical intervention. Commonality between these COSs may suggest a role for developing a paediatric COS that is applicable to all conditions with significant childhood morbidity and that can then be augmented with smaller disease-specific COSs. Results of two further paediatric surgical COSs that are currently in development for appendicitis^13^ and burns^14^ will help to inform this discussion, as will the results of the Core outcomes in neonatology (COIN) study,^15^ which is developing a neonatal COS.

While some of the outcomes included in the COS such as time on parenteral nutrition and number of operations were already frequently investigated in gastroschisis studies, there was significant variation in the way they were defined, or the time-point at which they were measured.^7^ This has meant that although researchers were investigating outcomes of importance to patients, the fact that they were doing so in different ways was impeding the development of a meaningful evidence base.^4^ Developing this COS has allowed key stakeholders to achieve consensus on definitions and measures that should be used for each of these already commonly investigated core outcomes. Promoting the use of these definitions will improve the quality of the evidence base supporting the management of infants with gastroschisis, without significantly altering the outcomes that researchers are investigating. Other core outcomes including growth and quality of life were very infrequently investigated in gastroschisis studies.^7^ It is likely that these have not previously been frequently investigated, because it is difficult and expensive to collect data in relation to them. Identifying these outcomes as important to key stakeholders therefore has significant implications for researchers, journal editors and funders, as it will alter the way in which studies are designed, funded and reviewed for publication.

While the COS has identified eight outcomes that, because of their importance to key stakeholders, should be investigated in all studies comparing treatments for children born with gastroschisis, there are still further steps that must be taken before the patient benefit of this work is realised. Using the COS in clinical practice, audit, observational studies and randomised controlled trials will start to establish data in the public domain that can be meta-analysed to meaningfully inform the ongoing debate around the ideal management of children born with gastroschisis. If this COS facilitates the generation of high-quality evidence to support optimal management strategy, then patient care can be standardised, and outcomes will begin to improve.

**Table 6:** Comparison of median phase one scores for outcomes included in the core outcome set between participants in each panel who completed all three phases of the Delphi process and those who only completed phase one

Outcome	Panel	P value from Wilcoxon rank-sum test
Death	Neonatal	0.3
	Non-neonatal	1
	Personal experience	0.9
Sepsis	Neonatal	0.4
	Non-neonatal	0.7
	Personal experience	0.7
Growth	Neonatal	0.4
	Non-neonatal	0.6
	Personal experience	0.2
Number of operations	Neonatal	0.9
	Non-neonatal	1
	Personal experience	0.2
Severe gastrointestinal complication	Neonatal	0.6
	Non-neonatal	0.6
	Personal experience	0.5
Time on parenteral nutrition	Neonatal	0.8
	Non-neonatal	0.7
	Personal experience	0.6
Liver disease	Neonatal	0.5
	Non-neonatal	0.9
	Personal experience	0.1
Quality of life	Neonatal	0.8
	Non-neonatal	0.4
	Personal experience	0.4
