# Supplementary material for: Development of a gastroschisis core outcome set
Source: Arch Dis Child Fetal Neonatal Ed. 2018 Mar 14;104(1):F76–82. doi: 10.1136/archdischild-2017-314560 (PMC6762000; doi:10.1136/archdischild-2017-314560)
Supplement: Supplementary file 1 [file fetalneonatal-2017-314560supp001.pdf]

Supplementary material 1 – Outcomes assessed in Delphi process

Red = outcomes from systematic review one
Blue = outcomes from systematic review two
Black = merged common term

Time to first enteral feed
Time to first oral feed
Time to full enteral feed
Time to full oral feeds
Incidence of PN
Parenteral nutrition ever required
Duration of PN
Time on parenteral nutrition
Time on ***total*** parenteral nutrition
Need for PN after discharge
Parenteral nutrition required post-discharge
Feeding, initiation of feed in NICU
Feeding, full feeds at discharge from NICU
Short gut syndrome
Short Bowel Syndrome
Bowel lengthening procedure required
Liver transplantation
Neurodevelopmental outcome
Neurodevelopmental delay
Developmental milestones >6m
Ever ventilated?
Incidence of ventilation
Post-operative ventilation required
Duration of respiratory support
Total time on mechanical ventilation
Duration of ventilation
Ventilated beyond 24hr
Post closure time on mechanical ventilation
Ventilation, peak inspiratory pressure
Ventilation, peak concentration inspired oxygen
Need for O2 after Discharge
Duration of O2
Respiratory compromise
Diagnosis of RDS
Neonatal Respiratory Distress Syndrome
Cholestasis

Time to first enteral feed
Time to full enteral feeds
Parenteral nutrition ever required
Time on parenteral nutrition
Time on ***total*** parenteral nutrition
Need for PN post discharge
Feeding, initiation of feed in NICU
Feeding, full feeds at discharge from NICU
Short bowel syndrome
Bowel lengthening procedure required
Liver transplantation
Neurodevelopmental outcomes
Developmental milestones >6m
Ventilation ever required
Post-operative ventilation required
Duration of respiratory support
Duration of ventilation
Ventilated beyond 24hr
Post closure time on mechanical ventilation
Ventilation, peak inspiratory pressure
Ventilation, peak concentration inspired oxygen
Need for O2 after Discharge
Duration of O2
Respiratory compromise
Respiratory distress syndrome
Cholestasis

Hypothyroidism	Hypothyroidism
Bacteraemia	Bacteraemia
pH, time acidotic	pH, time acidotic
Kidney dysfunction	Kidney dysfunction
Urine output	Urine output
Volume of IV fluid required	Volume of IV fluid required
Blood pressure, mean arterial	Blood pressure, mean arterial
Need for stoma	Need for stoma
Bowel Resection	Bowel Resection
Need for mesh at closure	Need for mesh at closure
Growth outcome	Unspecified measures of growth
Weight gain	
Weight<10th centile	Weight<10th centile
Length of Hospital Stay	Length of hospital stay
Length of stay	
NICU length of stay	NICU length of stay
Discharge, NICU to home	Discharge, NICU to home
Total number of GA	Total number of GA
General anaesthesia, number of days, indication	General anaesthesia, number of days, indication
Central-line usage ratio (days with central line/hospital days)	Central-line usage ratio (days with central line/hospital days)
Duration of antibiotics	Duration of antibiotics
Hospital charge	Hospital charge
Days to abdominal wall closure	Days to abdominal wall closure
Re-hospitalisation	Re-hospitalisation
Infectious complications	Unspecified infection
Infection, unspecified or other	
CVC sepsis	Central line related infections
Infection, central line related	
Wound infection or breakdown	Wound infection or breakdown
Infection with systemic sequelae	Infection with systemic sequelae
Infection free survival	Infection free survival
Infection, urinary or respiratory	Infection, urinary or respiratory
Transfusion	Transfusions
Number of transfusions	
Silo Complication	Silo Complication
Bowel ischaemia	Bowel ischaemia
Ischaemic bowel	
Anastomotic stricture	Anastomotic Stricture
Anastomotic stricture	
Perforation	Intestinal perforation
Intestinal perforation	
Intra-abdominal pressure	Intra-abdominal pressure
Abdominal compartment syndrome	Abdominal compartment syndrome

NEC<br>NEC	NEC
Stoma complication	Stoma complication
Obstruction<br>Adhesional small bowel obstruction	Bowel obstruction
TPN liver disease<br>Intestinal Failure Associated Liver Disease	Intestinal failure associated liver disease
Re-operation<br>Unplanned surgery<br>Unplanned reoperation	Re-operation
Reoperation, need for enlargement of gastroschisis defect	Reoperation, need for enlargement of gastroschisis defect
Reoperation, need for silo replacement	Reoperation, need for silo replacement
Ventral hernia<br>Umbilical hernia	Ventral hernia
GI complication	GI complication
Non-GI complication	Non-GI complication
Retinopathy of prematurity	Retinopathy of prematurity#
Presence of peel	Presence of peel
Gestational Age	Gestational Age
C-section	C-section
Birth weight	Birth weight
Birth weight below 2500g	Birth weight below 2500g
APGAR at 5M	APGAR at 5M
APGAR at 10M	APGAR at 10M
Neonatal convulsions	Neonatal convulsions
QT interval	QT interval
Simple vs. complex	Simple vs. complex
Birth Related Injury	Birth Related Injury
Cord pH<7.1 at birth	Cord pH<7.1 at birth
Cosmesis	Cosmesis
New outcome added in phase two following stakeholder nomination	Quality of life for the child
New outcome added in phase two following stakeholder nomination	Chronic gastrointestinal symptoms
New outcome added in phase two following stakeholder nomination	Gastrointestinal dysfunction, including but not limited to constipation
New outcome added in phase two following stakeholder nomination	Small bowel transplant
New outcome added in phase two following stakeholder nomination	Meningitis
New outcome added in phase two following stakeholder nomination	Mortality*
New outcome added in phase two following stakeholder nomination	Full oral feed achieved without any mechanical assistance

New outcome added in phase two following stakeholder nomination	Total number of days with a central line
New outcome added in phase two following stakeholder nomination	Discharge home with mechanical feeding assistance
New outcome added in phase two following stakeholder nomination	Societal costs, including financial cost for the family
New outcome added in phase two following stakeholder nomination	Home ***total*** parenteral nutrition
Existing outcome from phase one modified for phase two	Need for stoma split into "need for a permanent stoma" and "Need for temporary stoma"

Did not meet criteria for assessment in phase three of the Delphi process
*Inadvertently omitted from phase one of the Delphi process due to an error in transcription of outcomes from the reviews to the Delphi software
